# Regulation of Keratin Chemical Modifications: Potential Molecular Mechanisms in Proliferative Diseases

**DOI:** 10.3390/ijms27020972

**Published:** 2026-01-19

**Authors:** Xuemei Ma, Xiaoli Jiang, Mengxue Song, Bingbing Bai, Xia Hou, Qingtian Wu

**Affiliations:** 1 Department of Basic Medicine, Jiamusi University, Jiamusi 154007, China; 18645482322@163.com (X.M.); 15966512196@163.com (X.J.); 18845053812@163.com (B.B.); 2 Department of Clinical Medicine, Jiamusi University, Jiamusi 154007, China; 18212685516@163.com

**Keywords:** keratin, protein modification, fibrosis and cancer, epithelial cells

## Abstract

Keratin, a core structural protein in epithelial cells, is essential for maintaining epithelial tissue integrity. Numerous studies have confirmed its critical role in proliferative disorders, including lung/liver cancer, idiopathic pulmonary fibrosis (IPF), and hepatic fibrosis (HF). Post-translational modification (PTM) regulates protein activity, and keratin undergoes phosphorylation, acetylation, and methylation—modifications that modulate fibrosis and cancer progression by regulating relevant signaling pathways. However, how these modifications reshape keratin’s structure and function in these diseases remains understudied, underscoring the necessity for a systematic review. This review first summarizes keratin’s classification, physiological functions, and roles in epithelial cells, then focuses on the physiological significance of keratin modifications in fibrosis and cancer, while dissecting the molecular mechanisms by which keratin PTMs drive disease progression to address the knowledge gap regarding modification-related keratin changes. Elucidating the mechanisms of keratin and its PTMs is pivotal for understanding disease progression and developing targeted therapies; meanwhile, keratin-targeted strategies—such as keratin siRNAs and small-molecule compounds that regulate keratin expression or modification—have shown therapeutic potential. In summary, this review synthesizes current research findings and provides novel insights for the treatment of fibrosis and cancer.

## 1. Introduction

Fibrosis is a group of diseases characterized by excessive deposition of scar tissue in organs, which leads to structural damage and loss of function, and is closely associated with massive deposition of extracellular matrix [1,2]. Currently, most researchers believe that fibrosis is mainly caused by environmental pollution, viral infections or autoimmune diseases under the combined stimulation of multiple factors, the result of abnormal repair of organ damage [3,4]. Prolonged stimulation by pathogenic factors leading to progression of fibrosis to cancer is closely related to aberrant activation of signaling pathways with alterations in key proteins [5,6,7,8]. Such disorders have a significant impact on the normal physiological function of organs and may potentially jeopardize human life. Fibrosis has long been recognized as an independent risk factor for cancer development, as well as a substantial worldwide healthcare burden, due to its rising incidence and lack of effective therapies [9,10,11]. However, the complex molecular mechanisms involved in fibrosis and cancer processes have not been elucidated. Recently, the association of keratin chemical modification with disease and its potential as an emerging therapeutic target has received widespread attention [12,13,14].

Bill Astbury discovered keratin, a fibrous protein, in wool, horn, nails, and muscle in 1934. It serves as a cytoskeletal protein, and accurate regulation of post-translational modifications and keratin-associated proteins is crucial for maintaining normal cellular physiological functions. Mutations in keratins cause post-translational changes (PTMs) of keratins and related proteins, resulting in diseases [13,15]. In recent years, the role of keratin in various diseases has been widely studied [16,17]. However, despite these research areas being comprehensively summarized, the PTMs involved in keratins have been seldom specifically discussed in the literature, and the specific mechanisms by which they are implicated in disease are largely unknown.

PTMs are modifications of specific amino acid residues in target proteins that determine the complexity and diversity of protein functions. Proteins undergo a myriad of PTMs and thus accurate cellular regulation, including phosphorylation, acetylation, methylation, glycosylation, O-GlcNAcylation, oxidative modifications, and other modifications [18]. They regulate protein function through rapid activation, inhibition, or sustained degradation, thereby participating in a wide range of physiological processes, of which phosphorylation was discovered in 1906 and was the first PTM to be studied. It acts as a double-edged sword, promoting or hindering the degradation of misfolded proteins to maintain states of health and disease. Increasing evidence suggests that chemical modifications of related proteins of the keratin family, such as Keratin 8 (K8), Keratin 18 (K18), and Keratin 19 (K19), play important roles in regulating fibrosis and cancer disease [19,20,21,22]. Regulation of key protein interactions or signaling pathways through direct or indirect processes that are central to disease development. Therefore, further elucidation of the regulatory functions of keratin modifications may provide new avenues for clinical intervention in fibrosis and cancer. This review outlines the molecular structure and functions of keratin, reviews the mechanisms of chemical modification, and elucidates the significance of keratin modification in fibrosis and cancer progression. It also discusses the siRNA targeting keratin for in vivo therapy, further exploring whether this regulatory mechanism could be a potential therapeutic measure for fibrosis and cancer.

## 2. Molecular Structure and Physiological Function of Keratin

To date, 54 functional keratin genes have been identified in humans, which are classified into two subfamilies (Type I: acidic; Type II: neutral or basic) based on the acidity and alkalinity of their amino acid sequences [23]. (see Table 1). The two are co-assembled into functional units in a 1:1 ratio by heterodimerization [24]. Keratins are classified as hard (α-keratins) or soft (β-keratins) based on their physical properties [25,26,27,28]. The expression of keratin genes exhibits high tissue specificity. Genes from different subgroups exert specific functions in different epithelial tissues and physiological/pathological processes, The core functions of different keratin subtypes are shown in Table 2 [29,30,31,32,33,34].

### 2.1. Molecular Structure of Keratin

**Figure 1:**
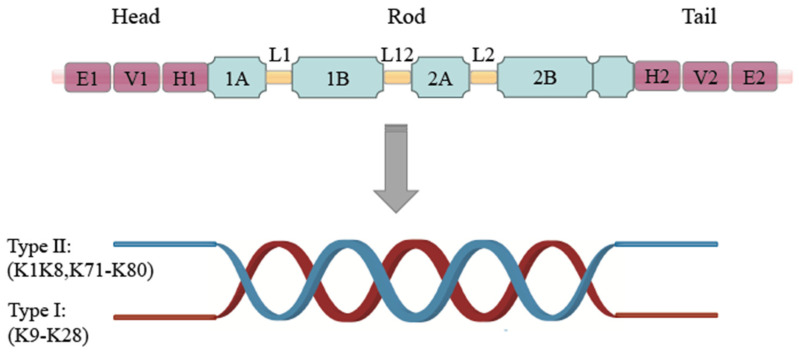
Schematic illustration of intermediate filament protein structure and dimer assembly. The same color represents the same type of protein. Top: Monomeric intermediate filament protein, comprising Head, Rod, and Tail domains with subdomains (E1, V1, H1, 1A, 1B, L1, L12, 2A, 2B, H2, V2, E2). Bottom: Protein dimerization process: Two α-helical segments (Type I: K9–K28; Type II: K1K8, K71–K80) in the Rod domain twist together to form a coiled-coil dimer.

Epithelial keratins are the main components that make up intermediate fiber (IF) proteins and play a crucial role in maintaining cellular and tissue integrity [35,36]. The hierarchical assembly of keratins from monomers to dimers to tetramers to protofibers and finally winding into intermediate fibers is highly ordered. A keratin monomer is a polypeptide chain consisting of about 400–600 amino acid residues, mainly comprising an N-terminal head structural domain (rich in glycine and serine), a central rod region, and a C-terminal tail structural domain (rich in cysteine). One of the central rod regions consists of about 310 amino acids and contains the highly conserved central α-helical rod-like structural domain [37,38,39,40]. The formation of the α-helix is mainly dependent on the heptapeptide repeat sequence, which is the periodic arrangement of every seven amino acids (heptad repeats in the pattern a-b-c-d-e-f-g), where hydrophobic residues located at the a- and d-positions promote hydrophobic interactions between the helices. This region is further divided into helical segments 1A, 1B, 2A, and 2B, separated by short-chain non-helical linkage regions (L1, L12, and L2). Subsequently, the α-helices of the rod region of the two monomers are entangled with each other through hydrophobic interactions and hydrogen bonding to form a left-handed superhelical structure, which forms a dimer by heterologous pairing of type I and type II keratins [39,40,41]. Two heterodimers were formed in reverse parallel to generate a high-tensile tetramer. The tetramers were then stacked longitudinally and transversely to generate protofibers with a diameter of about 10 nm. Intermediate fibers with a diameter of about 10–12 nm were eventually formed by helically entangled cross-linking of eight protofibers (Figure 1). This structure could make keratin a key molecule for protective barriers and structural support. Keratins’ molecular structure influences the variety of protein activities, and minor variations in keratin structure may result in significant illnesses [42]. For example, the p.Arg125Cys mutation in Keratin 14 (K14) disrupts the convoluted helical structure, leading to epidermal relaxation blisters [43,44]. Mutations in keratin genes (e.g., K5/K14) may impair fiber assembly, triggering a series of pathological changes such as increased cellular fragility and tissue damage.

### 2.2. Potential Physiological Functions of Keratin

The molecular structure of keratins determines their potential physiological functions. Keratin is the major structural protein of the vertebrate epidermis and its appendages, and intermediate filaments (IFs) serve as a major component of the cytoskeleton to maintain structural stability, tissue- and cell-specific functions, and protection against external stimuli [45,46]. Previous research has shown that keratin is largely involved in providing mechanical support and barrier integrity in cells and tissues [47]. However, in recent years, it has been shown that keratins serve critical roles in non-classical physiological processes such as immunological modulation, metabolic regulation and signaling [14,20,48]. This also raises the following question: are all keratins beneficial for physiologically relevant functions?

#### 2.2.1. Keratin Is Directly Involved in Immune Regulation and Inflammatory Modulation

**Figure 2:**
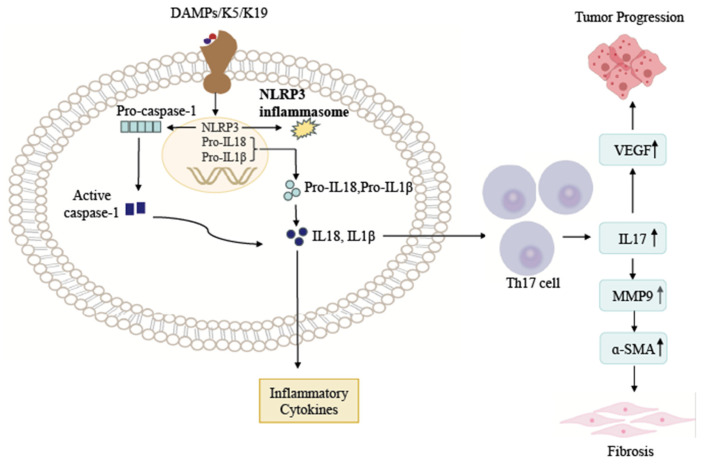
Schematic of DAMPs/K5/K19-induced NLRP3 inflammasome activation and Th17 cell-mediated tumor progression and fibrosis. Trigger: The binding of DAMPs, K5, and K19 to their cognate receptor proteins triggers activation of the NLRP3 inflammasome. Inflammasome-mediated cytokine maturation: Pro-caspase-1 is activated (cleaved) to caspase-1, which processes pro-IL18/pro-IL1β into mature IL18/IL1β; these cytokines are released as inflammatory mediators. Downstream effects: Secreted IL18/IL1β act on Th17 cells, driving upregulation of IL17, MMP9, VEGF, and α-SMA—subsequently promoting tumor progression and fibrosis.

Studies have shown that keratin activates chemokine expression through interactions with heterogeneous nuclear ribonucleoprotein K (hnRNPK) and autoimmune regulator (AIRE), promoting recruitment and activation of inflammatory cells, thereby exacerbating local inflammatory responses [49,50]. Meanwhile, keratin can act as a ligand to recognize apoptotic or necrotic cells in a pH-dependent manner and participate in the clearance of abnormal cells by the immune system (Figure 2). The discovery implies that keratins may play a fundamental physiological role in inducing inflammation formation as well as the immune response [30,48,51].

#### 2.2.2. Keratin Modulates Energy Metabolism

Keratin-mediated regulation of metabolic homeostasis has emerged as a novel study area in recent years [52,53,54]. The “physiological role” of keratin in this process remains largely unknown. Keratin stimulates protein synthesis and speeds up tissue regeneration during the skin wound healing process [55,56].

#### 2.2.3. Bidirectional Action of Keratin

Keratins in tumors may interact with other proteins to trigger downstream signaling pathways and increase cancer cell invasiveness [57,58]. Also, keratins are stably expressed in malignant transformation, and fragments in K18 and K19 serum serve as key indicators of fibrosis and tumor progression [59,60]. Furthermore, keratin plays a crucial function in various disorders, for instance, K5 and K14 mutations cause epidermolysis bullosa simplex (EBS), which may be a direct result of altered cytoskeleton by basal cell lysis [61]; epidermoproliferative palmoplantar keratosis (EPPK) is caused by K9-specific mutations; and K2e mutations expressed only in the intestinal epithelium lead to irritable bowel syndrome (IBS). Furthermore, nonclassical functions of keratins are also involved in the development of disease. Hyperphosphorylation of K8 and K18 ultimately contributes to the deterioration of human liver disease [62]. It is clear that its own mutations and interactions with cellular components directly or indirectly affect the pathways of cell proliferation, differentiation, and death, thereby leading to disease, from the liver to the kidney to the lung.

## 3. Role of Keratin in Epithelial Cells

Keratin is a key molecule that dynamically regulates cellular function, and its expression pattern is closely related to epithelial type. In vitro, keratin is a fundamental structural component of the outer layer of skin, fingernails, and toenails; in vivo, keratin is found in epithelial cells to maintain cytoskeletal integrity and metabolic activity [60,63,64,65]. In conclusion, there is growing evidence that keratin plays a crucial role in various epithelial-related diseases. The main manifestations are:

### 3.1. Intestinal Epithelium

The folds of the crypts of the colon and the finger villi and crypts in the small intestine of mammals consist of a single layer of columnar epithelial cells that secrete a mucus layer that protects the intestinal epithelium from mechanical injury [66,67]. The intestinal epithelium is the core executive unit of intestinal function, and its structural integrity and dynamic balance depend on the synergistic effects of multiple molecular mechanisms [68]. Keratin expression follows a similar pattern across the epithelium, with K8 and K19 being the most often expressed keratins in the human colonic epithelium, with K18 appearing in minor amounts [69]. Keratin expression levels in the intestinal epithelium varied depending on localization and degree of cellular differentiation; for instance, K18 was most strongly expressed in cup cells in the upper part of the villi in the lower part of the crypts, whereas K20 was weakly expressed at the bottom of the colon’s crypts but increased in differentiated luminal cells. In addition to this, it was found that K8 was expressed most differently at different sites. Researchers have speculated that K8 expression may be related to inter-individual differences as well as intracryptic grading, which aptly illustrates the potential activity and impact of the intestinal keratin profile [16].

**Figure 3:**
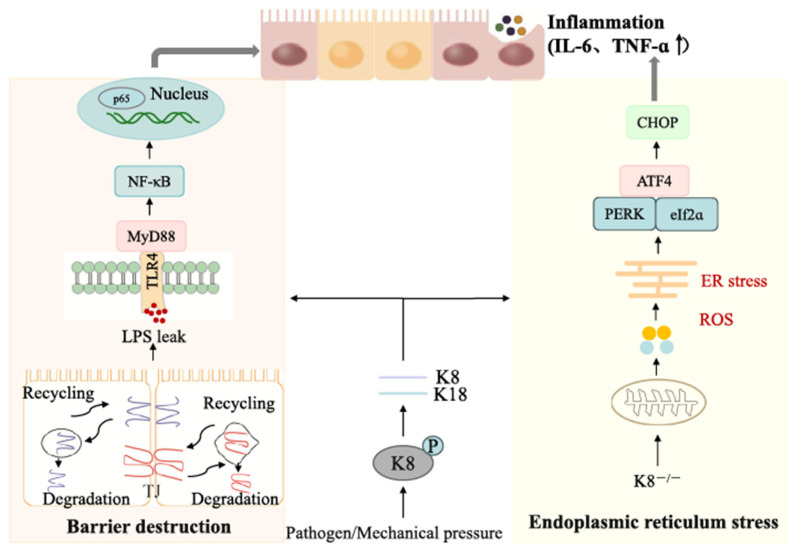
Schematic of K8/K18-mediated regulation of barrier destruction, inflammation, and endoplasmic reticulum (ER) stress induced by pathogen/mechanical pressure. Trigger: Pathogen/mechanical pressure acts on keratin K8 (K8^0−^ = modified/abnormal K8), initiating downstream cascades. Barrier destruction and inflammation (**left**): Impaired K8/K18 recycling/degradation disrupts the barrier, causing LPS leakage. LPS-TLR4 binding activates the NF-κB pathway (via MyD88); nuclear-translocated p65 drives secretion of inflammatory cytokines (IL-6, TNF-α). ER stress (**right**): Inflammation/stimuli induce ER stress (activated via the PERK/eIF2α/ATF4/CHOP axis) accompanied by reactive oxygen species (ROS) production; K8^0−^ regulates this ER stress process.

Recent data indicates that keratin expression in the intestinal epithelium may be directly related to inflammation and metabolism [48]. Keratin is a key node in the interaction between inflammation and metabolism by modulating immune responses and metabolic pathways. In the colonic mucosa of K8−/− mice, decreased monocarboxylate transporter 1 (MCT1) expression, reduced 3-hydroxy-3-methylglutaryl-CoA synthase 2 (HMGCS2) activity, and higher short-chain fatty acids (SCFA) levels in the feces were observed. This is because colon cells rely on meta-produced butyrate for energy through beta-oxidation and glycolysis [70]. K8−/− mice had altered gut microbiota and metabolism, which contributed to intestinal inflammation (Figure 3). This shows that keratins play a significant role in the complicated networks of inflammation and metabolism. Thus, normal keratin expression provides support for epithelial cell stability and integrity, whereas aberrant expression causes a variety of epithelial cell diseases.

### 3.2. Urinary Tract Epithelium

The uroepithelium forms tight junctions and a urine–blood barrier with complex signaling functions [71]. Urothelial epithelial cells differentiate into three layers: basal, intermediate, and superficial umbrella cells. They inhibit urea, poisons, and pathogens in urine from penetrating deeper tissues through tight junctions and a lipid layer of asymmetric unit membranes (AUPs) [72,73]. In vivo, the bladder urinary tract epithelium’s proliferation index remained around 0.01% and was nearly quiescent. Nevertheless, basal stem cells are able to proliferate and differentiate rapidly after injury to repair damaged epithelium. In the uroepithelium, keratin expression is highly stratified and specific, such as K5/K14 in the basal layer, K7/K8/K18/K19 in the middle layer, and K20 in the superficial layer [74,75,76].

Keratin 5 is a major structural protein of the basal uroepithelium, and K5 uroepithelial cells (K5-UCs) of the kidney proliferate at varying rates throughout development. It has been demonstrated that K5-UCs contribute to the repair of damaged urinary tract epithelium and function as superior progenitor cells. Fibroblast growth factor 7 (FGF7) is critical for rescuing adult K5-UC progenitor cell activity [77,78,79]. As a result, when the K5 mutation leads to the inability to polymerize with K14 properly, the intermediate fiber network breaks down, and the basal cells lose the ability to resist stretching, leading to epithelial stratification disorders triggering uroepithelial leakage, chronic inflammation, and cancer [80]. Keratin is not only the “structural scaffold” of the urinary epithelium but also a multifunctional molecule that dynamically regulates barrier function, regeneration, and disease progression. Through multidisciplinary intersections, keratin research will provide new perspectives for the mechanistic analysis and therapeutic strategies of urological diseases.

### 3.3. Respiratory Epithelium

The respiratory epithelium is the epithelial tissue that covers the surface of the respiratory system, and its primary function is commonly thought to be a physical barrier [81,82]. From the nasal cavity to the alveoli, the airway epithelium evolves from a complex squamous epithelium to a pseudocomplex ciliated columnar epithelium and eventually to a single layer of flat epithelium. With the extension of the airways into the lungs, there are differences in the type of cellular composition [83]. Brush cells are scattered in the epithelium, and the dense microvilli on their surface form synaptic connections with nerve endings at the base, which may act as chemoreceptors to monitor changes in the airway’s internal environment and trigger the cough reflex to clear irritants [84,85]. Neuroendocrine cells, on the other hand, regulate local immunomodulation and vascular tone by secreting active substances such as 5-hydroxytryptamine and calcitonin gene-related peptide (CGRP). The airway epithelium not only serves as a physical barrier and clearance system, but it also maintains respiratory homeostasis through the coordinated action of different cell types [86].

Keratins, the predominant family of intermediate fibrous proteins in epithelial cells, play multiple critical roles in respiratory epithelium [87,88]. In order to repair the damaged region of the respiratory tract caused by smoke or infection, basal cells use a heterodimer made up of K5 and K14 to relay signals that trigger cell growth and differentiation into ciliated or secretory cells [89]. In addition, the secretory activity of cup cells is also regulated by keratin. K19 modulates the synthesis and vesicular transport of the mucin by influencing the morphology of the endoplasmic reticulum and Golgi apparatus; its aberrant expression may result in excessive mucus production or altered viscoelasticity, notably in the pathogenesis of chronic obstructive pulmonary disease (COPD) and lung cancer [90,91]. These roles demonstrate that keratins are not just static structural proteins but also the core of multifunctional molecular networks dynamically involved in the maintenance of respiratory homeostasis and disease regulation.

## 4. The Role of Keratin Modifications in Proliferative Diseases

In mammalian cells, proper folding and PTM of keratins are essential for normal development and maintenance of homeostasis in the organism [92,93]. Phosphorylation, acetylation, ubiquitination, SUMOization, and glycosylation are the primary forms. PTM usually alter the structure, function, and localization of proteins by changing amino acid side chains [94,95]. It enables cells to make relevant modifications in response to external stimuli in a short period of time, much faster than gene transcription and protein synthesis. Simultaneous modification of the dual properties of enzymes (acetylase/deacetylase, kinase/phosphatase) allows cells to flexibly switch between activation and inhibition, ensuring a rigorous cell cycle [96,97,98]. Furthermore, chemical modifications enable the same protein to perform multiple functions in different states of modification, and these factors work together to regulate protein properties and structure, forming complex and diverse signaling systems that regulate cellular processes associated with the pathophysiology of fibrosis and cancer [99,100]. Such as immunological evasion, metabolic reprogramming, and DNA damage and repair. Keratin modification has been less studied in proliferative diseases, and this section mainly summarizes its association with liver fibrosis, liver cancer, colorectal cancer, and psoriasis.

### 4.1. Keratin Phosphorylation and Liver Fibrosis

Protein phosphorylation is a reversible PTM process [101]. Phosphate bonds are created when γ-phosphate groups from ATP are transferred to certain amino acid residues (serine, threonine, or tyrosine) of proteins. Phosphatases are then in charge of removing the phosphate groups [102,103,104]. In the human genome, phosphorylation activities are regulated by around 568 protein kinases and 156 protein phosphatases [105,106]. The catalytic structural domain of protein kinase consists of a typical structural domain and key residues, with the N-terminal leaflet, the C-terminal leaflet, and the catalytic cleft dominating the typical structure, and the key residues, in the catalytic loop, Asp and Mg^2+^ ions stabilizing the ATP phosphate group [107,108]. Through the utilization of distal binding domains, protein kinases can improve substrate selectivity and particularly identify common sequences in substrates [109]. Protein phosphatases are classified into three families: phosphoprotein phosphatase (PPP), metal-dependent protein phosphatase (PPM), and protein tyrosine phosphatase (PTP) [110]. Protein phosphorylation is a critical step in signal transmission, metabolic regulation, and cell cycle control by dynamically modifying the activity, interaction, and localization of proteins [111,112,113,114]. As a result, mutations in protein phosphate sites are connected with the onset and development of a variety of diseases.

Liver fibrosis is primarily a wound-healing response to chronic viral or metabolic liver damage, which eventually progresses to cirrhosis or even cancer [115,116]. The absence of effective therapeutic therapy has failed to significantly slow its progression, putting an enormous strain on families and society. Liver fibrosis is characterized by excessive extracellular matrix (ECM) accumulation and activation of hepatic stellate cells (HSCs) [117,118]. Collectively, these alterations affect cell signaling and function, directly affecting cellular structure and eventually leading to liver fibrosis. Despite an array of clinical medication studies targeting pathologic characteristics, their efficacy has often been lower than predicted, pushing researchers to investigate alternative possible causes of liver fibrosis [119,120]. One such discovery is that keratin phosphorylation is closely related to the pathological process of liver fibrosis [121].

**Figure 4:**
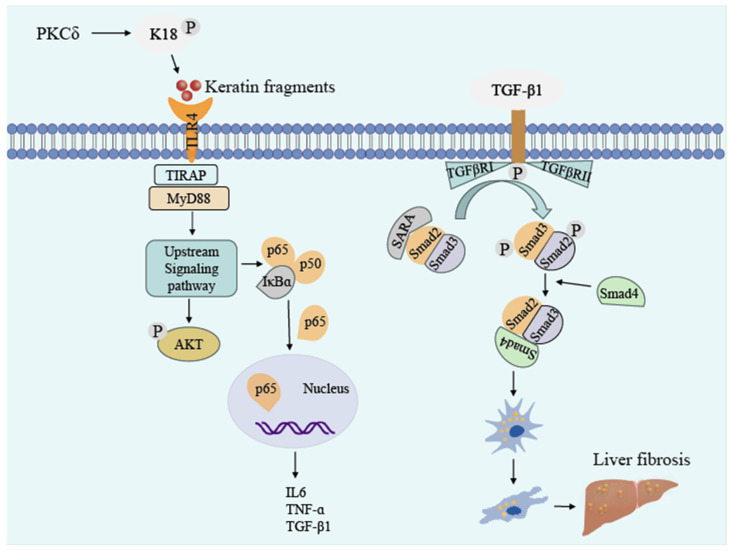
Schematic of PKCδ-mediated K18 phosphorylation regulating inflammatory pathways and the TGF-β1/Smad axis in hepatic fibrosis. “P” stands for phosphorylation. Upstream initiation: PKCδ phosphorylates K18 to generate keratin fragments. These fragments bind to TLR4, activating upstream signaling via TIRAP/MyD88—subsequently triggering AKT activation and NF-κB (p65/p50) dissociation from IκB. Cytokine induction: Nuclear-translocated p65 drives the expression of inflammatory mediators (IL-6, TNF-α) and TGF-β1. Fibrosis progression: TGF-β1 binds to its receptors (TGFβRI/II), inducing Smad phosphorylation; the formed Smad complex (Smad2/3/4) translocates into the nucleus, ultimately promoting hepatic fibrosis.

Keratin 8/18 (K8/K18) is a key hepatocyte protective protein [122]. Patients with liver illness have abnormal K8 and K18 expression [123,124,125,126]. Studies have shown that K8 and K18 mutants cause liver injury and accelerate hepatocyte apoptosis [127]. In general, K8S74 phosphorylation protects the liver from harm by decreasing SAPK activity on other substrates [20]. When the K8Gly62-Cys (Gly62 replaced by Cys, K8G62C) mutation alters cellular conformation, it produces an imbalance in the regulation of SAPKs by K8S74, hastening apoptosis in hepatocytes [128]. Furthermore, following hepatocyte damage, cellular stress-activated protein kinase (PKCδ) phosphorylates particular serine sites of K18 [129]. This reduces the assembly ability of keratin intermediate filaments, causing the cytoskeletal network to disintegrate. Depolymerized keratin fragments are released extracellularly and recognized by macrophages as damage-associated molecular patterns (DAMPs). This activates the Toll-like receptor (TLR4) signaling pathway and promotes the secretion of IL-6, TNF-α-associated inflammatory factors, and TGF-β1 profibrotic factors [130,131]. TGF-β1 induces the conversion of hepatic stellate cells into activated myofibroblasts through the Smad-dependent pathway. These myofibroblasts release high amounts of type I collagen (collagen 1), resulting in a fibrous scar (Figure 4). IL-10 has a function in down-regulating inflammatory cytokines [132,133]. In a clinical study of a treatment cohort using IL10, keratin phosphorylation was reduced in approximately 70% of patients, while levels were elevated or unchanged in 30%. The therapeutic impact of IL10 was also discovered to be connected to long-term keratin phosphorylation while failing to correct short-term induced phosphorylation changes [134]. Given that there have been no serious side effects reported with IL-10, it is regarded as a promising candidate for the development of new therapeutic strategies for liver disease [135].

### 4.2. Keratin Phosphorylation and Liver Cancer

Primary liver cancer is a malignant tumor that develops in the liver and is extremely aggressive and fatal [136,137]. Globally, liver cancer is the sixth-most common cancer and the third leading cause of cancer-related deaths. Hepatocellular carcinoma (HCC) accounts for 75–85% of primary liver cancers. Patients are frequently diagnosed with advanced liver cancer due to a lack of early detection methods. Despite ongoing advances in the treatment of hepatocellular carcinoma (surgical resection, radiation, chemotherapy, interventional therapy, and targeted therapy), the 5-year survival rate for individuals with advanced hepatocellular carcinoma is less than 12% [138,139,140,141]. In recent years, researchers discovered that abnormal cytokeratin 19 (CK19) modification is directly linked to the development of hepatocellular carcinoma [142].

CK19 is mostly found in intrahepatic bile duct epithelial cells and hepatic progenitor cells. Normal mature hepatocytes do not express CK19; nonetheless, it may be abnormally expressed in chronic liver disease, liver fibrosis, and hepatocellular cancer [143,144]. CK19 expression in hepatocellular carcinoma cells indicates a bad prognosis [145]. Laminin-332 and epidermal growth factor were demonstrated to independently promote CK19 expression in hepatocellular carcinoma cells. JNK/SAPK phosphorylation may stimulate the EGF-EGFR signaling pathway, which contributes to the development of CK19+ HCC [146]. It was also shown that the long non-coding RNA KILH (Linc-KILH) interacts with CK19 and stimulates phosphorylation of CK19 at the Ser 35 position, causing CK19 to restructure from a filamentous to a granular form and undergo membrane translocation, promoting hepatocellular cancer [147]. On the other hand, Linc-KILH enhances the interaction between β-catenin and CK19 in the cytoplasm, promoting its nuclear translocation [148]. Furthermore, the researchers discovered that Revafenib was more effective in treating CK19+ HCC than CK19− [149,150]. This suggests that CK19 might be a viable treatment target for HCC.

### 4.3. Keratin Acetylation and Colorectal Cancer

Acetylation was first found in 1963 and has since been intensively researched. It influences gene expression, metabolic control, and cellular stress responses by modifying protein charge, conformation, and interactions [151]. This process is primarily dependent on histone acetyltransferases (HATs), which transfer the acetyl group from acetyl-CoA to target proteins, and histone deacetylases (HDACs) or NAD+-dependent deacetylases (sirtuins), which remove the acetyl group to regulate the process [152,153]. There is substantial evidence that HATs, HDACs, and acetyl-lysine-binding proteins are involved in cellular functions other than the regulation of transcription mechanisms [154]. Furthermore, acetylation interacts with other post-translational changes to produce complex regulatory networks such as phosphorylation–acetylation synergy or antagonism, methylation–acetylation exclusion, and acetylation–ubiquitination competition [17,155,156]. Consider the p53 protein as an example. After DNA damage, ATM/ATR kinase phosphorylates p53, inducing a conformational shift and exposing lysine residues, allowing acetylation by acetyl transferase and increasing p53 DNA binding capacity. Under certain settings, certain phosphorylation events may recruit deacetylases to remove acetyl groups, inhibiting p53 activity and establishing a dynamic equilibrium [157]. Acetylation has a wide range of effects on the function of cytoskeletal proteins in addition to regulating gene expression [158]. However, the specific role of keratin acetylation in tumorigenesis and tumor progression remains to be further explored.

Colorectal cancer is the most common malignant tumor of the digestive tract worldwide [159]. Approximately 10% of patients have a family history of colorectal cancer, and inflammatory bowel illness and intestinal polyps both enhance the risk of cancer. The prevalence of colorectal cancer in China is increasing, with a median 5-year survival rate of 57.6%. Surgery is the primary curative treatment for patients in the early stages of the disease, with a 5-year survival rate of over 90% for stage I patients [160]. However, tumor metastasis and drug resistance remain the main causes of treatment failure [161]. The link between keratin acetylation and colorectal cancer is gradually gaining attention [162]. This is mostly expressed in two ways. On the one hand, aberrant keratin expression correlates with the invasiveness of colorectal cancer. On the other hand, post-translational keratin alteration may influence tumor metastasis by controlling cytoskeletal dynamics.

K8 and K18 are type II cytokeratins that are widely expressed in the human colonic mucosa [163]. Acetylation of K8 and K18 has a tight relationship with intestinal mucosa nutrition, metabolism, and function [69,164]. The study discovered that K18 expression levels were higher in colon cancer cell lines, owing mostly to the involvement of the CBP (P300) protein in controlling the acetylation process, which in turn regulates K18 promoter activity [165]. This demonstrates that CBP regulates acetylation, which affects K18 expression in colon cancer. Keratin acetylation, as a fundamental link between cellular structural control and tumor metabolism, has the potential to open up new avenues for precision colorectal cancer treatment [166,167]. However, its function and specific mechanism in colorectal cancer have not been thoroughly studied. However, its function and specific mechanism in colorectal cancer have not been thoroughly studied.

### 4.4. Chemical Modification of Keratin and Psoriasis

FUT11 (fucosyltransferase 11) is highly expressed in psoriatic lesions, which catalyzes the fucosylation modification of keratin 17 (K17). Fucosylation enhances the interaction between K17 and the E3 ubiquitin ligase Trim21, thereby promoting K63-linked ubiquitination (a type of non-degradative ubiquitination). K63-linked ubiquitination significantly increases the protein stability of K17, enabling it to sustainedly activate the AKT/mTOR signaling pathway and drive the unlimited proliferation of keratinocytes. The FUT11-K17-Trim21 axis can serve as a novel therapeutic target for psoriasis, and inhibition of FUT11 or Trim21 is expected to block K17-mediated abnormal proliferation [168].

## 5. Conclusions and Future Prospects

Over the last decade, studies on post-translational keratin changes (such as phosphorylation, acetylation, and ubiquitination) have gradually revealed its complicated regulation network in proliferative illnesses (such as organ fibrosis and tumor proliferation). For example, an imbalance in ubiquitin-mediated keratin degradation has been linked to epithelial–mesenchymal transition (EMT) and fibrosis development, and inhibitors targeting ubiquitin ligases have been shown to inhibit abnormal keratin in preclinical models. However, when these strategies are translated into clinical practice, their efficacy is often limited by tissue-specific differences or genetic heterogeneity. PTM, as a central regulatory mechanism of cellular homeostasis, has a direct function in the etiology of proliferative disorders by disrupting its dynamic balance. Recent research has discovered that PTM plays an important role in the remodeling of the fibrotic milieu or tumor cell invasion by precisely regulating the stability and function of keratin and its interacting proteins. However, there are still considerable gaps in studies on the keratin modification pathway. First, whether the key enzymes in the modification pathway have selective regulatory effects in specific tissues (such as the liver and lungs) undergoing fibrosis or tumor formation, and whether their molecular mechanisms are specific to disease type or pathological stage, still need to be further explored; Second, it is unclear if phosphorylation, ubiquitination, and acetylation constitute synergistic or antagonistic regulation, influencing keratin’s mechanical characteristics or signal transduction capabilities; Third, while previous research has identified key regulatory enzymes that control keratin homeostasis and developed small-molecule inhibitors targeting their active sites, the selectivity and long-term safety of these compounds in complex pathological microenvironments require further validation using more sophisticated preclinical models; Fourth, the keratin modification network may influence disease development by multi-target cascade effects, but the specifics of this multidimensional regulation and its pathological aspects are unknown.

In conclusion, keratin chemical modification is critical in proliferative disorders like fibrosis and cancer. The discovery of small interfering RNA in vivo applications has provided new directions and possibilities for the treatment of fibrosis and cancer. Furthermore, combining single-cell epigenomics with mechanical signal imaging technology to better understand the dynamic regulation of keratin modification in tissue hardness perception and mechanical signal transduction will aid in the development of novel therapeutic strategies based on the “force-chemical decoupling” mechanism. With advancements in gene editing technology and organoid models, it will be possible to reveal the causal relationship between the keratin modification network and the progression of proliferative diseases at higher spatio-temporal resolutions and to translate basic research findings into precision treatment methods targeting the remodeling of the fibrotic microenvironment or the breakthrough of tumor metastasis barriers.

## Figures and Tables

**Table 1:** Classification of human functional keratin genes.

Gene Type	Main Members	Chromosomal Localization
Type I (Acidic)	Epithelial type: K9~K20 (including K14, K15, K16, K17, etc.); Hair and follicle-specific type: K25~K28, K31~K40 (11 hair keratins in total)	Mostly located at 17q21; K18 is exceptionally located at chromosome 12
Type II (Neutral/Alkaline)	Epithelial type: K1~K8 (including K6A~C, etc.); Hair and follicle-specific type: K71~K86 (6 hair keratins in total)	Concentrated at 12q13

**Table 2:** Biological functions of keratin genes.

Specific Gene (Type I/II)	Core Functions
Type I: K1, K5, K14, K15	Provides cellular mechanical integrity and maintains epithelial tissue structural homeostasis
Type I: K16, K17 Type II: K6B~C, K6A	Serves as a biomarker for activated keratinocytes
Type II: K6A	Enhances tumor cell migration and invasion, and acts as an independent prognostic indicator for colorectal cancer
Type I: K13, K19	participates in scar formation by regulating actin cytoskeleton organization and inflammatory responses
Type II: K3	Mutations cause Meesmann corneal dystrophy
Type I: K25~K28, K31~K40 Type II: K72~K74, K76~K86, K6irs1~K6irs4	Key genes for hair formation and structural stability; Inner root sheath-specific K25~K28/K6irs1~K6irs4 participate in inner root sheath structure construction and ensure normal hair growth and shedding cycles
Type II: K71	Affects hair fiber characteristics by regulating hair follicle development
Type II: K75	Deletion causes curly feather traits in chickens

## Data Availability

No new data were created or analyzed in this study. Data sharing is not applicable to this article.

## Abbreviations

AIRE	autoimmune regulator
ATR	ataxia-telangiectasia and Rad3-related
ATM	ataxia-telangiectasia mutated
AUPs	asymmetric unit membranes
CBP	CREB-binding protein
COPD	chronic obstructive pulmonary disease
CK19	cytokeratin 19
DAMPs	damage-associated molecular patterns
EGF	epidermal growth factor
EGFR	epidermal growth factor receptor
ECM	extracellular matrix
EMT	epithelial–mesenchymal transition
EBS	epidermolysis bullosa simplex
EPPK	epidermoproliferative palmoplantar keratosis
FUT11	fucosyltransferase 11
FGF7	Fibroblast growth factor 7
hnRNPK	heterogeneous nuclear ribonucleoprotein K
HATs	histone acetyltransferases
HDACs	histone deacetylases
HCC	hepatocellular carcinoma
HMGCS2	3-hydroxy-3-methylglutaryl-CoA synthase 2
HSCs	hepatic stellate cells
IF	intermediate fiber
IBS	irritable bowel syndrome
IL-6	interleukin-6
IL-10	interleukin-10
JNK	c-Jun N-terminal kinase
K1	Keratin 1
K5	Keratin 5
K8	Keratin 8
K9	Keratin 9
K14	Keratin 14
K18	Keratin 18
K19	Keratin 19
K2e	Keratin 2e
KILH	long non-coding RNA KILH (Linc-KILH)
L1	linkage region 1
L12	linkage region 12
L2	linkage region 2
MCT1	Monocarboxylate transporter 1
PKCδ	protein kinase C delta
PPP	phosphoprotein phosphatase
PPM	metal-dependent protein phosphatase
PTMs	post-translational modifications
PTP	protein tyrosine phosphatase
SAPK	stress-activated protein kinase
SCFA	short-chain fatty acids
siRNA	small interfering RNA
TGF-β1	transforming growth factor-beta 1
TLR4	Toll-like receptor 4
TNF-α	tumor necrosis factor-alpha
